# The role of Src & ERK1/2 kinases in inspiratory resistive breathing induced acute lung injury and inflammation

**DOI:** 10.1186/s12931-017-0694-7

**Published:** 2017-12-13

**Authors:** Dimitrios Toumpanakis, Vyronia Vassilakopoulou, Ioanna Sigala, Panagiotis Zacharatos, Ioanna Vraila, Vassiliki Karavana, Stamatios Theocharis, Theodoros Vassilakopoulos

**Affiliations:** 1 1st Department of Critical Care and Pulmonary Medicine and “Marianthi Simou” Applied Biomedical Research and Training Center, Medical School, University of Athens, 45-47 Ispilandou str, 10676 Athens, Greece; 2 Department of Pathology, Medical School, University of Athens, Athens, Greece

**Keywords:** Src, ERK1/2, Resistive breathing, Lung injury

## Abstract

**Background:**

Inspiratory resistive breathing (IRB), a hallmark of obstructive airway diseases, is associated with large negative intrathoracic pressures, due to strenuous contractions of the inspiratory muscles. IRB is shown to induce lung injury in previously healthy animals. Src is a multifunctional kinase that is activated in the lung by mechanical stress. ERK1/2 kinase is a downstream target of Src. We hypothesized that Src is activated in the lung during IRB, mediates ERK1/2 activation and IRB-induced lung injury.

**Methods:**

Anaesthetized, tracheostomized adult rats breathed spontaneously through a 2-way non-rebreathing valve. Resistance was added to the inspiratory port to provide a peak tidal inspiratory pressure of 50% of maximum (inspiratory resistive breathing). Activation of Src and ERK1/2 in the lung was estimated during IRB. Following 6 h of IRB, respiratory system mechanics were measured by the forced oscillation technique and bronchoalveolar lavage (BAL) was performed to measure total and differential cell count and total protein levels. IL-1b and MIP-2a protein levels were measured in lung tissue samples. Wet lung weight to total body weight was measured and Evans blue dye extravasation was estimated to measure lung permeability. Lung injury was evaluated by histology. The Src inhibitor, PP-2 or the inhibitor of ERK1/2 activation, PD98059 was administrated 30 min prior to IRB.

**Results:**

Src kinase was activated 30 min after the initiation of IRB. Src inhibition ameliorated the increase in BAL cellularity after 6 h IRB, but not the increase of IL-1β and MIP-2a in the lung. The increase in BAL total protein and lung injury score were not affected. The increase in tissue elasticity was partly inhibited. Src inhibition blocked ERK1/2 activation at 3 but not at 6 h of IRB. ERK1/2 inhibition ameliorated the increase in BAL cellularity after 6 h of IRB, blocked the increase of IL-1β and returned Evans blue extravasation and wet lung weight to control values. BAL total protein and the increase in elasticity were partially affected. ERK1/2 inhibition did not significantly change total lung injury score compared to 6 h IRB.

**Conclusions:**

Src and ERK1/2 are activated in the lung following IRB and participate in IRB-induced lung injury.

## Background

Resistive breathing is the hallmark of diseases of airway obstruction, such as upper airway obstruction, asthma and COPD, especially during exacerbations and/or stable severe disease [1]. Resistive breathing is associated with large negative intrathoracic pressures, due to strenuous contractions of the inspiratory muscles, especially the diaphragm. Increased mechanical stress is an injurious stimulus for the lung [2], resulting in lung injury through stress membrane failure [3] and/or induction of intracellular pathways (i.e. mechanotransduction [4, 5]). Indeed, our research group has shown that inspiratory resistive breathing induces acute lung injury in previous healthy rats [6] and resistive breathing through tracheal banding provokes pulmonary inflammation in mice [7]. Moreover, the effect of resistive breathing-induced lung injury is dose dependent, since the greater the load imposed during respiration, the more pronounced the subsequent lung injury [8]. However, the mechanism of IRB-induced lung injury remains largely unknown.

The non-receptor tyrosine kinase Src plays an essential role in endothelial permeability regulation and inflammatory processess [9]. Src is a multifunctional kinase that is activated early upon mechanical stretch [10]. Following activation, Src signaling includes downstream molecules that regulate the cell phenotype. Extracellular regulated kinase (ERK1/2) activation by a Src-mediated mechanism has been reported in vitro following stretch of lung epithelial cells [11]. We have previously shown that Src kinase is activated early in the lung following inspiratory resistive breathing and ERK1/2 is activated at a latter time point [6]. In an ex vivo murine model of ventilator-induced lung injury, inhibition of the Src kinase attenuated the increase in capillary permeability [12]. Pretreatment with a Src kinase inhibitor also prevented the LPS-induced lung inflammation, by reducing the expression of pro-inflammatory cytokines (e.g. TNFa) and preventing neutrophil influx into the lungs [13]. To the extent of our knowledge, the contribution of Src kinase activation to resistive breathing-induced acute lung injury has never been studied.

We hypothesized that Src kinase activation during resistive breathing contributes to IRB-induced lung injury. We also hypothesized that ERK1/2 is activated secondary to Src kinase activation during IRB and also participates in IRB-induced lung injury. To test our hypothesis, a model of inspiratory resistive breathing in previously healthy rats has been employed [6, 14], while Src and ERK kinase involvement was studied with pretreament with the spesific Src inhibitor, PP-2 and the inhibitor of ERK1/2 activation, PD98059, respectively.

## Methods

### Subjects

Adult female rats (age 8–12 weeks, weight 230 ± 30 g) were used in this study (see figure legends for the number of animals in each experimental group). Animals were purchased from the Hellenic Pasteur Institute and were housed in a 12-h day/night cycle at the Experimental Surgery Unit of Evangelismos Hospital being provided with food and water ad libitum.

### Inspiratory resistive breathing (IRB) model

A model of IRB was set, as previously described [6, 14]. Briefly, rats were anaesthetized with an intraperitoneal (ip) injection of a mixture of ketamine (75 mg/kg) and xylazine (5 mg/kg) and tracheostomized (tracheal cannula, 14G). After a short stabilization period (~15 min), the tracheal cannula was connected to a two-way non-rebreathing valve (Hans-Rudolf). With the use of a pressure transducer, the tracheal pressure was monitored and the maximum inspiratory pressure (Pi,max) was measured during spontaneous breathing efforts through a totally occluded inspiratory port for 10 s (Direcwin, Raytech Instruments Inc.). Then, the inspiratory port was connected to a tube of small diameter (resistance) and the diameter was adjusted to provide a peak tidal inspiratory pressure (Pi) at 50% of maximum (inspiratory resistive breathing, IRB), to mimic severe airway obstruction, as seen in COPD exacerbation and asthma attacks [6]. The animals were randomly assigned to 6 h of IRB. During the procedure the inspiratory port was connected to a 100% oxygen supply to prevent hypoxemia. Spontaneously breathing animals that breathed 100% oxygen against no load for equal time point, served as controls. Supplemental doses of ketamine (30 mg/kg, ip) were given during the procedure, as needed.

### Src and ERK1/2 inhibition

In a subgroup of animals, the Src inhibitor PP-2 (Calbiochem) was administered 30 min prior to IRB at a dose of 1 mg/kg ip in 5% DMSO [9]. ERK1/2 activation was inhibited by pretreatment with PD98059 (1 mg/kg ip) in 5% DMSO (Calbiochem) [15]. Control animals and 6 h IRB group (without the inhibitor) received only 5% DMSO ip. The efficacy of the aforementioned doses to inhibit kinase activation was evaluated by western blot analysis (see below).

### Respiratory system mechanics

Following 6 h of either resistive or quietly breathing, the animals were further anaesthetized with an ip injection of ketamine (75 mg/kg) and xylazine (10 mg/kg), the two-way non-rebreathing valve was removed and the tracheal cannula was connected to a small animal ventilator (Scireq, Montreal, Canada). Succinylcholine (8 mg/kg ip) was administrated, to cease spontaneous breathing. During ventilation, heart rate was monitored to ensure adequate depth of anaesthesia. Prior to measurements (30 s) the lung volume history was once standardized by simply occluding the expiratory line of the ventilator until the airway opening pressure reached 30 cmH_2_O. The impedance of the respiratory system was then measured by the forced oscillation technique (FOT). Impedance (Z) was then fitted to constant phase model: Zrs(f) = Rn + i2πfI + (G-iH)/(2πf)^a^, where Rn is the newtonian resistance of the airways, i is the imaginary unit, f is the frequency, I is the inertance of the gas in the airways, G represents tissue damping (a parameter closely related to tissue resistance that reflects the energy dissipation in the alveoli), H represents tissue elasticity and alpha can be calculated through the equation α = (2/π)arctan(H/G).

### Bronchoalveolar lavage (BAL)

Following lung mechanics measurements, the animals were sacrificed by exsanguination (vena cava dissection) under anaesthesia and the thoracic cavity was exposed. The left lung was temporarily ligated and the right lung was lavaged with 3 aliquots of 2.5 ml normal saline. BAL fluid was withdrawn and immediately centrifuged (300 g × 10 min, 4 °C). The supernatant was stored at −80 °C. The cell pellet was resuspended to 1 ml of normal saline.

### Total protein in BAL fluid

Total protein concentration of BAL fluid was estimated, as an indirect index of capillary protein leakage (lung permeability), using a colorimetric protein assay according to manufacturer (BioRad, USA). Bovine serum albumin was used to create standard curves.

### Cell count (Total – Differential)

Total cell counts were performed and aliquots (4 × 10^4^ cells/slide) were pelleted on glass slides by cytocentrifugation. Differential counts were performed on May-Grűnwald-stained cytospins and percentages of macrophages, monocytes, lymphocytes and eosinophils/basophils were determined by counting their number in 300 cells. Eosinophil/basophil count was negligible and was omitted from further analysis.

### Evans blue dye extravasation

In a different group of animals treated with PD98059, permeability of pulmonary vasculature following 6 h of inspiratory resistive breathing was measured using the Evans blue dye extravasation technique, as previously described [6]. At the end of the IRB session, animals were re-anaesthetized and 40 mg/kg of Evans blue was injected in the femoral vein. The animals were connected to the ventilator, ventilated for a total of 40 min and then were sacrificed. The lungs were removed from the thoracic cavity by excluding the trachea and mainstem bronchi. Evans blue was extracted from pulmonary tissues after homogenization in 1 ml of normal saline. This volume was added to 2 volumes of deionized formamide and incubated at 60 °C for 18 h. After centrifugation at 2000 *g* for 30 min the supernatant was collected. Evans blue in the lung tissue was quantitated by dual wavelength spectrophotometric analysis at 620and 740 nm. This method corrects the specimen’s absorbance at 620 nm for the absorbance of contaminating heme pigments, using the following formula: corrected absorbance at 620 nm = actual absorbance at 620 nm – [1.426*(absorbance at 740) + 0.03]. We calculated a permeability index from the corrected pulmonary tissue Evans blue absorbance at 620 nm, normalized to the total body weight.

### Gravimetric parameters

In a subgroup of animals after resistive breathing, the left lung was immediately removed and weighed. The ratio of wet lung weight to total body weight was measured, as an index for the presence of pulmonary edema [16].

### Protein extraction from lung tissue samples

Following BAL, the right main bronchus was ligated and the right lung was removed, immediately frozen by immersion in liquid nitrogen and stored at −80 °C for further analysis. Frozen lung sections were homogenized with buffer containing 50 mM Hepes (pH 7.5), 150 nM NaCl, 10% glycerol, 1% Triton X-100, 1 mM EDTA, 1.5 mM MgCl_2_ and a cocktail of protease and phosphatase inhibitors at a 1:1000 concentration. The samples were then centrifugated at 10.000 x g for 10 min. The supernatant was the collected and total protein concentration was estimated using a colorimetric protein assay according to manufacturer (BioRad, USA).

### Western blot analysis

Lung tissue homogenate samples were separated on a 10% SDS-polyacrylamide gel. Proteins were then electrophoretically transferred onto PVDF membrane and blocked for 1 h at room temperature with 5% non fat milk in Tris-buffered saline containing 0.2% Tween (TBS-T). The membranes were then incubated with an anti-p-Src kinase (Tyr416, 1:500, #2101, Cell Signalling), an anti-t-Src (1:1000, #2110, Cell Signaling), an anti-p-ERK1/2 (Thr202/Tyr204, 1:500, #9101, Cell Signaling) or an anti-t-ERK1/2 (1:500, #9102, Cell Signaling) antibody overnight at 4 °C. Membranes were probed with anti-rabbit or anti-mouse secondary antibodies (Jackson Immunoresearch Lab.) for 2 h at room temperature. Antibody labelling of protein bands was detected with enhanced chemiluminescence (ECL) reagents according to the supplier’s protocol. To normalize for protein loading, membranes were probed with anti-actin antibody (MAB1501, Chemicon Int.). Band intensity was quantified by Gel Pro software.

### Cytokine levels in lung tissue samples

The protein levels of IL-1β, a proinflammatory cytokine that has been shown repeatedly to be upregulated following resistive breathing in the lung [6, 8, 17], and MIP-2a (a central neutrophilic chemotactic factor [18]) were measured with ELISA from lung tissue homogenates samples with the standard protocol supplied by the manufacturer (DuoSet ELISA, R&D Systems, Minneapolis, USA). The protein levels of IL-1b and MIP-2a were normalized for total protein content of tissue homogenate samples and were expressed as pg/mg of lung tissue [6].

### Lung histology

The left lung was fixed with 4% formaldehyde under 20 cmH_2_O pressure and removed. After 24 h the lung tissue was embedded in paraffin using conventional techniques and cut in serial 5-μm sections. Sections were stained with haematoxylin and eosin. A lung injury score was determined based on the following histological features: (i) focal alveolar membrane thickening, (ii) capillary congestion, (iii) intra-alveolar haemorrhage, (iv) interstitial and (v) intra-alveolar neutrophil infiltration. Each feature was scored from 0 to 3 based on its absence (0) or presence to a mild (1), moderate (2), or severe (3) degree [19].

### Statistical analysis

*D*ata are presented as mean ± standard error of the mean (SEM). Statistical analysis was performed with one way analysis of variance (ANOVA). When significant, post hoc analysis was performed with the Fisher’s LSD test. A *p* value <0.05 was chosen, as statistically significant. Data from pathology were ordinal and were analyzed with the non-parametric Kruskal-Wallis ANOVA and Mann-Whitney U test for post hoc analysis.

## Results

### Src kinase activation during IRB

**Fig. 1:**
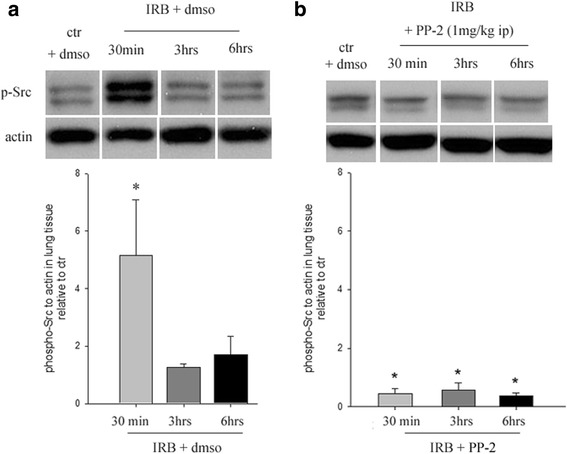
Representative Western blot analysis of phosphorylated (p)-Src kinase at Tyr416 in lung tissue samples during IRB and the efficacy of the Src kinase inhibitor PP-2 (1 mg/kg ip) (representative western blot analysis, *upper,* band intensity quantification, *lower*). **a**. The ratio of p-Src to actin is elevated at 30 min of IRB, while returns to control levels after 3 and 6 h of IRB. Quantification of band intensities revealed a ~5× fold upregulation of p-Src to actin at 30 min of IRB (**b**). PP-2 administration inhibited Src phosphorylation during the entire IRB session. Data presented as mean ± SEM, *n* = 4–13 per group, *p < 0.05 to ctr, grey bars, 30 min IRB, dark grey bars, 3 h IRB, black bars, 6 h IRB

In accordance with our previous study [6], IRB was associated with an early activation of Src through phosphorylation at Tyr416. The p-Src to actin ratio increased nearly 5-fold relatively to control levels at 30 min IRB (*p* = 0.002) and returned to baseline at 3 h and 6 h of IRB (Fig. 1a). When the Src inhibitor PP-2 was administered prior to IRB (1 mg/kg ip), the increase of p-Src to actin at 30 min was ameliorated. The inhibition of Src was sustained for the total duration of IRB (Fig. 1b). IRB did not influence total Src expression at any time point (t-Src to actin ratio relative to ctr, 30 min IRB: 1.18 ± 0.41, 3 h IRB: 1.06 ± 0.08, 6 h IRB: 1.11 ± 0.09, ANOVA F = 0.139, *p* = 0.936).

### The effect of Src kinase inhibition on IRB-induced pulmonary inflammation

**Fig. 2:**
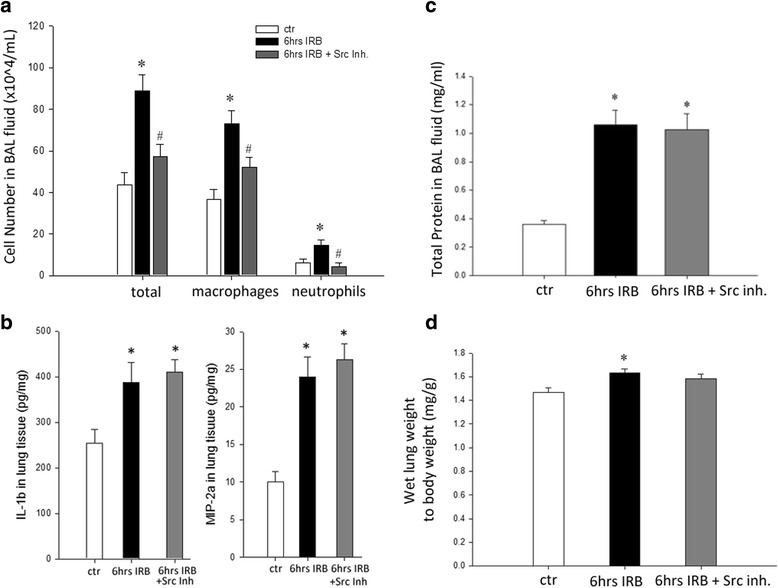
Effect of Src kinase inhibition on IRB-induced lung injury. **a**. Src inhibition attenuated the increase in BAL cellularity, by reducing the count of both macrophages and neutrophils, following 6 h of IRB. **b**. 6 h of IRB was followed by an increase in lung tissue levels of both IL-1β *(left)* and MIP-2a *(right)* relative to control, an effect that was not influenced by Src inhibition. **c**. PP-2 administration prior to IRB did not alter the increase in lung permeability, as assessed by total protein levels in BAL fluid. **d**. Wet lung to total body weight, an index of pulmonary edema, was increased after 6 h of IRB, a finding not affected by PP-2 administration. Data presented as mean ± SEM, *n* = 5–16 per group, *p < 0.05 to ctr, #*p* < 0.05 to 6 h IRB, white bars, control, black bars, 6 h IRB, dark grey bars, 6 h IRB + Src Inh

Following 6 h of IRB an increase in BAL cellularity was documented (~2× fold to ctr, *p* < 0.001), due to increased numbers of both macrophages and neutrophils (p < 0.001 and *p* = 0.005, respectively). PP-2 administration inhibited the increase in total and differential cell numbers (6 h IRB + Src Inh: total, *p* = 0.002, macrophages *p* = 0.012 and neutrophils *p* = 0.0014 to 6 h IRB, see Fig. 2a), although there was a trend for macrophages to remain elevated compared to control values (*p* = 0.064). Lymphocyte count was not altered after 6 h IRB (ANOVA, F = 1.371, *p* = 0.27). 6 h of IRB were associated with increased protein levels of the pro-inflammatory cytokines IL-1β and MIP-2a, in lung tissue samples (IL-1β, p = 0.012 to ctr, MIP-2a, *p* < 0.001 to ctr). PP-2 had no effect on IL-1β and MIP-2α expression in lung tissue following 6 h of IRB (Fig. 2b).

### The effect of Src kinase inhibition on lung permeability and gravimetric parameters following 6 h of IRB

After 6 h of IRB an increase in total protein levels in BAL fluid was found compared to ctr (*p* < 0.001), an indirect index of increased alveolar-capillary membrane permeability. Also, the wet lung weight to body weight ratio was increased at 6 h of IRB (*p* = 0.001 to ctr), suggesting the presence of pulmonary edema. Total protein levels in BAL fluid remained significantly elevated after PP-2 administration, compared to ctr values (6 h IRB + Src Inh.: total protein in BALf, p < 0.001 to ctr, Fig. 2c). PP-2 administration had no effect on increased wet lung to body weight ratio at 6 h of IRB (Fig. 2d).

### The effect of Src kinase inhibition on respiratory system mechanical derangement following 6 h of IRB

**Fig. 3:**
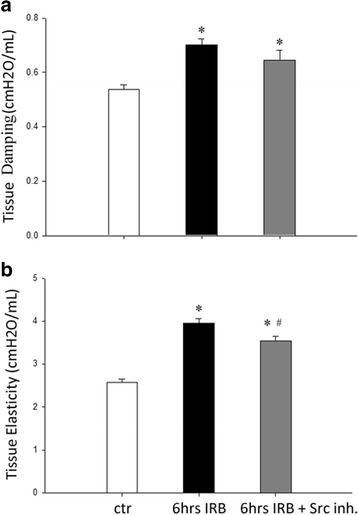
Respiratory system mechanics following 6 h IRB and the effect of PP-2 pretreatment. **a**. Following 6 h of IRB a significant increase in tissue damping was measured by the forced oscillation technique that was not affected by Src inhibition. In contrast, PP-2 pretreatment partially blunted the increase of tissue elasticity, after 6 h of IRB, although it remained significantly changed, compared to control values (**b**). Data presented as mean ± SEM, *n* = 7–18 per group, *p < 0.05 to ctr, #*p* < 0.05 to 6 h IRB, white bars, control, black bars, 6 h IRB, dark grey bars, 6 h IRB + Src Inh

IRB resulted in a significant derangement of respiratory system mechanics. FOT revealed an increase in tissue damping and elasticity at 6 h of IRB, compared to ctr (*p* < 0.001 and p < 0.001, Fig. 3a and b, respectively and Table 1). Pretreatment with the Src inhibitor PP-2 had a moderate effect on respiratory system mechanics alterations. In details, following Src inhibition, tissue elasticity was reduced following 6 h IRB (*p* = 0.01, Fig. 3b), although it did not return to control values. Tissue damping increase following 6 h IRB was not affected by PP-2 pretreatment (Fig. 3a). Interestingly, a significant increase of airway resistance was measured at 6 h IRB, compared to ctr (Rn-6 h IRB, *p* = 0.009 to ctr), that was not affected by Src inhibition (Table 1).

**Table 1:** Mechanical parameters of the respiratory system following inspiratory resistive breathing and the effect of Src and ERK1/2 inhibition

	Ctr	6 h IRB	6 h IRB + Src Inh.	6 h IRB + ERK inh.
Rn - Airway Resistance (cmH2O.s/mL)	0.048 ± 0.001	0.055 ± 0.002 *	0.050 ± 0.002	0.051 + 0.001
G - Tissue Damping (cmH2O/mL)	0.53 ± 0.019	0.70 ± 0.02 *	0.64 ± 0.03 *	0.64 ± 0.02 *
H - Tissue Elasticity (cmH2O/mL)	2.57 ± 0.07	3.95 ± 0.10 *	3.53 ± 0.11 *, #	3.67 ± 0.06 *, #

Data presented as mean ± SEM, *n* = 7–18 per group. * *p* < 0.05 to ctr, # *p* < 0.05 to 6 h IRB

### Histological evaluation of lung tissue sections after Src inhibition

**Fig. 4:**
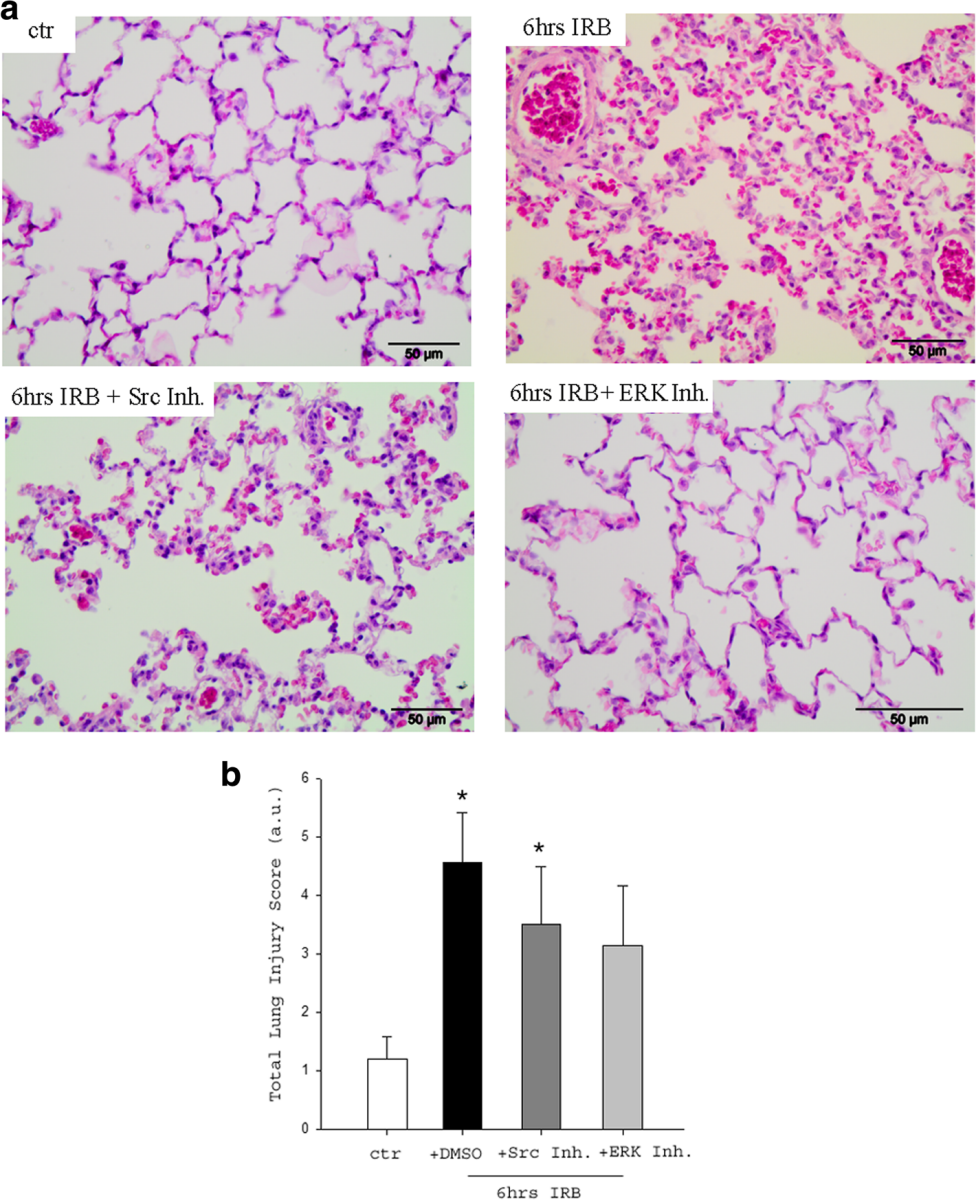
Histological analysis of lung tissue section following IRB and the effect of Src kinase and ERK inhibition. **a**. Representative H&E lung tissue sections of ctr (upper left), 6 h IRB (upper right), 6 h IRB + Src Inh. (lower left) and 6 h IRB + ERK Inh. (lower right). Note that after 6 h IRB, lung histology reveals features of lung injury, such as focal membrane thickening, capillary congestion and inflammatory cell infiltration. **b**. Quantification of lung injury in lung tissue sections confirmed the increased total lung injury score after 6 h IRB, compared to control. Neither after Src inhibition nor after ERK inhibition, there was a significant change compared to 6 h IRB. Data presented as mean ± SEM, *n* = 6–10 per group, * *p* < 0.05 to ctr, white bar, control, black bar, 6 h IRB, dark grey bar, 6 h IRB + Src Inh, grey bar, 6 h IRB + ERK Inh

Following 6 h of IRB, an elevated total lung injury score was found (*p* < 0.001 to ctr, Fig. 4), due to significantly increased focal thickening, capillary congestion, interstitial and intra-alveolar neutrophil infiltration (Table 2). Administration of the Src inhibitor PP-2 did not alter the increased total lung injury score at 6 h of IRB (6 h IRB + Src Inh. *p* = 0.022 to control, Fig. 4).

**Table 2:** Histological features of acute lung injury following inspiratory resistive breathing and the effect of Src and ERK inhibition

	Focal membrane thickening	Capillary congestion	Intra-alveolar hemorrhage	Interstitial neutrophil infiltration	Intra-alveolar neutrophil infiltration	Total lung injury score
Ctr	0.6 ± 0.16	0.3 ± 0.15	0.2 ± 0.13	0.1 ± 0.1	0 ± 0	1.2 ± 0.38
6 h IRB	1.33 ± 0.16*	1.22 ± 0.22*	0.55 ± 0.17	0.88 ± 0.26*	0.55 ± 0.17*	4.55 ± 0.85*
6 h IRB + Src Inh.	1.16 ± 0.16	1 ± 0.25*	0.33 ± 0.21	0.66 ± 0.33	0.33 ± 0.21	3.5 ± 0.99*
6 h IRB + ERK Inh.	0.85 ± 0.26	0.71 ± 0.18	0.57 ± 0.29	0.57 ± 0.29	0.42 ± 0.20	3.14 ± 1.01

Data presented as mean ± SEM, *n* = 6–10 per group. * *p* < 0.05 to ctr

### Downstream effect of Src inhibition on ERK1/2 activation in the lung during IRB

**Fig. 5:**
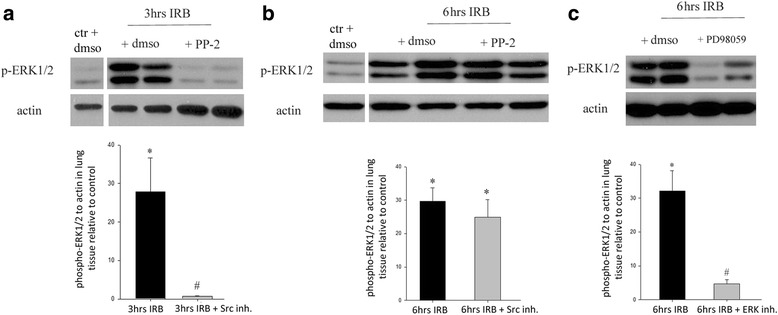
Activation of ERK1/2 through phosphorylation in lung tissue following IRB. The effect of upstream Src kinase inhibition and efficacy of the ERK activation inhibitor, PD98059 (representative western blot analysis, *upper,* band intensity quantification, *lower*). **a**. Following 3 h of IRB, activation of ERK was noticed in lung tissue samples (increased p-ERK to actin ratio relative to control), an effect that was blocked by Src inhibition. **b**. In contrast, ERK activation at 6 h of IRB was independent of Src kinase activation during IRB. **c** Administration of the inhibitor of ERK activation, PD98059 (1 mg/kg ip) prior to IRB, sufficiently attenuated the increase of p-ERK to actin ratio in lung tissue samples at 6 h IRB. Data presented as mean ± SEM, *n* = 3–6 per group for A, *n* = 6–9 per group for B and *n* = 6–8 per group for C, *p < 0.05 to ctr, #*p* < 0.05 to IRB equal time point, black bars, 6 h IRB, grey bars, 6 h IRB + Src Inh. **a** and **b** or 6 h IRB + ERK Inh. **c**

During IRB a significant activation through phosphorylation of ERK1/2 was found by western blot analysis. In details, at 3 h of IRB an almost 27-fold increase of p-ERK1/2 to actin ratio was measured in lung tissue samples, compared to control (*p* = 0.03, Fig. 5a). When the inhibitor of Src kinase, PP-2, was administered prior to IRB this effect was blunted (p = 0.02 to 3 h IRB, Fig. 5a). At 6 h of IRB, the p-ERK1/2 to actin ratio was also elevated (29-fold to control, *p* < 0.001). However, Src inhibition failed to attenuate ERK activation at 6 h of IRB (p < 0.001 to ctr, p non significant to 6 h IRB, Fig. 5b). 6 h of IRB did not influence expression of t-ERK, neither before nor after PP-2 administration (t-ERK1/2 to actin ratio relative to ctr, 6 h IRB: 1.29 ± 0.10, 6 h IRB + Src Inh.: 0.97 ± 0.17, ANOVA F = 1.141, *p* = 0.355, *n* = 4–6 per group). Administration of the inhibitor of ERK1/2 activation, PD98059, at a dose of 1 mg/kg ip 30 min prior to IRB initiation, inhibited the phosphorylation of ERK1/2 in lung tissue at 6 h of IRB (*p* < 0.001 to 6 h IRB, Fig. 5c).

### The effect of ERK1/2 inhibition on IRB-induced pulmonary inflammation

**Fig. 6:**
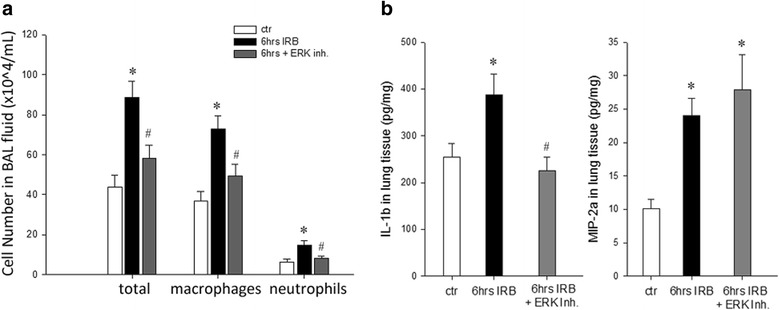
The effect of ERK inhibition on pulmonary inflammation induced by 6 h of IRB. **a**. Pretreatment with PD98059 inhibited the increase in total cell number in BAL fluid after 6 h of IRB, by reducing both macrophages and neutrophils count. **b**. ERK inhibition also abolished the increase in lung tissue protein levels of IL-1 *(left)*, while had no effect on the increased MIP-2a levels measured after 6 h of IRB *(right)*. Data presented as mean ± SEM, *n* = 10–11 per group for A, n = 5–15 per group for B. * p < 0.05 to ctr, # p < 0.05 to 6 h IRB, white bar, control, black bar, 6 h IRB, grey bar, 6 h IRB + ERK Inh

Inhibition of ERK activation ameliorated the increase of BAL cellularity, seen after 6 h IRB (*p* = 0.003 compared to 6 h IRB). Indeed, both macrophage and neutrophil counts returned to control values after PD98059 administration (*p* = 0.006 and *p* = 0.024 compared to 6 h IRB, respectively, Fig. 6a). Regarding the expression of inflammatory cytokines in lung tissue, ERK inhibition blocked the expression of IL-1β following 6 h IRB (*p* = 0.004 to 6 h IRB). In contrast, MIP-2a remained significantly elevated compared to control (MIP-2a: 2.77-fold to control, *p* = 0.001, Fig. 6b).

### The effect of ERK1/2 inhibition on lung permeability and gravimetric parameters following 6 h of IRB

**Fig. 7:**
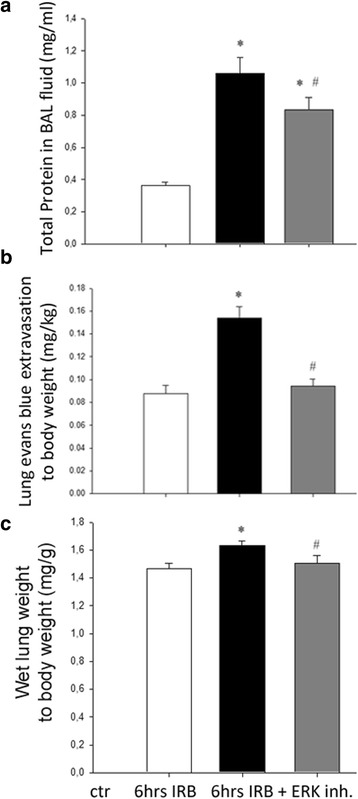
The effect of ERK inhibition on lung permeability and gravimetric parameters following 6 h IRB. **a**. ERK inhibition had a moderate effect on total protein level in BAL fluid, since although it remained significantly elevated to ctr, was also reduced compared to 6 h IRB. **b**. Pretreament with PD-98059 abolished the increase of evans blue dye extravasation in the lung at 6 h IRB, suggesting a protective effect of ERK inhibition on lung permeability. **c**. After ERK inhibition, the increase of wet lung weight to total body weight at 6 h IRB was blunted. Data presented as mean ± SEM, *n* = 13–16 per group for **a**, *n* = 4–8 per group for **b**, *n* = 10–11 per group for **c**. * *p* < 0.05 to ctr, # *p* < 0.05 to 6 h IRB, white bar, control, black bar, 6 h IRB, grey bar, 6 h IRB + ERK Inh

ERK inhibition partially inhibited the RB-induced increase of total protein level (6 h IRB + ERK inh: *p* < 0.001 to ctr, *p* = 0.049 to 6 h IRB, Fig. 7a). Since total protein level is an indirect index of lung permeability, alveolar-capillary membrane permeability at 6 h of IRB was also measured by the evans blue dye extravasation technique. Interestingly, ERK inhibition completely abolished the increase of evans blue dye extravasation at 6 h IRB (6 h IRB: p < 0.001 to ctr, p < 0.001 to 6 h IRB + ERK Inh., Fig. 7b). Furthermore, following PD98059 administration, the RB-induced increase of wet lung weight to total body weight was not observed at 6 h IRB (6 h IRB + ERK Inh.: *p* = 0.035 to 6 h IRB, Fig. 7c).

### The effect of ERK1/2 kinase inhibition on respiratory system mechanics derangement following 6 h of IRB

**Fig. 8:**
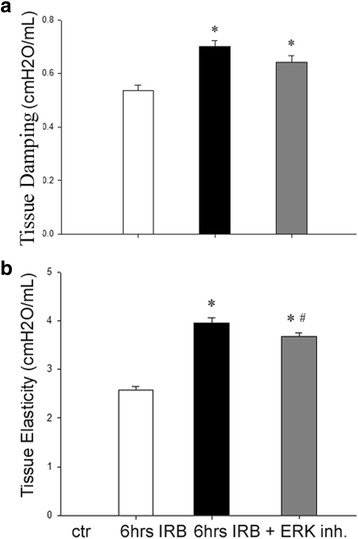
Respiratory system mechanics following 6 h IRB and the effect of PD98059 pretreatment. **a** ERK inhibition did not affect tissue damping increase after 6 h IRB. **b** In contrast, tissue elasticity was increased to a lesser degree at 6 h IRB, when ERK was inhibited. Data presented as mean ± SEM, *n* = 12–18 per group, *p < 0.05 to ctr, #*p* < 0.05 to 6 h IRB, white bars, control, black bars, 6 h IRB, grey bars, 6 h IRB + ERK Inh

Pretreatment with the inhibitor of ERK activation, PD98059, partially reversed respiratory system derangement at 6 h of IRB. More explicitly, after ERK inhibition, RB-induced increase in tissue elasticity was significantly reduced (*p* = 0.045 to 6 h IRB), although it remained elevated compared to control values (p < 0.001) (Fig. 8b). Tissue damping increase at 6 h IRB was not affected (6 h IRB + ERK Inh: *p* = 0.002 to control, Fig. 8a). As with SRC inhibition, airway resistance increase at 6 h IRB was not affected by ERK inhibition (Table 1).

### Histological evaluation of lung tissue sections after ERK inhibition

Following ERK inhibition, the total lung injury score at 6 h IRB was not altered (Fig. 4a lower right, b and Table 2).

## Discussion

The major findings of our study are: 1. Src kinase is early activated following initiation of IRB and inhibition of Src kinase activation ameliorates influx of inflammatory cells in alveolar spaces and has a moderate protective effect in IRB-induced derangement of lung mechanics, 2. During IRB, ERK is activated in lung tissue through an early Src-dependent and a late Src-independent mechanism, 3. ERK inhibition protects against IRB-induced lung injury.

Resistive breathing is the major pathophysiologic characteristic of diseases of airway obstruction. In both asthma and COPD, infiltration of airway wall with inflammatory cells, mucus secretion, airway wall remodeling and altered function/structure of airway smooth muscle cells lead to exaggerated airway obstruction, a phenomenon that is more pronounced in stable severe disease and especially during exacerbations [20, 21]. Resistive breathing is associated with increased negative intrathoracic pressure that acts as an injurious stimulus for the lung [1]. Our research group has previously shown that in previously healthy animals [both mice [7] and rats [6, 8]], resistive breathing induces acute lung injury. In details, as also shown in our current study, 6 h of inspiratory resistive breathing at 50% of Pi/Pi,max induced various features of lung injury, such as increased lung permeability, influx of leukocytes, upregulation of inflammatory cytokines and derangement of respiratory system mechanics.

The mechanisms that mediate the effects of resistive breathing on the respiratory system are unknown. Although the effects may be mediated by a direct “injurious” action of the mechanical force developed during resistive breathing [stress membrane failure model, introduced by West et al. [3]], it has also been shown that lung resident cells can “sense” the application of mechanical forces and adapt their function by activation of intracellular pathways [cellular mechanotransduction [4]]. The non-receptor tyrosine kinase Src is a multifunctional kinase that is found in vitro to be early activated through phosphorylation at Tyr416 upon mechanical stretch [10]. Indeed, only after 30 min of IRB, increased phosphorylated (Tyr-416) levels of Src were detected in lung tissue samples that then returned to control values. A similar pattern of Src activity was also documented in vitro in endothelial cells subjected to uni-axial cyclic stretch [22]. In vivo, high tidal volume ventilation is also associated with Src activation through phosphorylation [23].

To investigate the role of Src kinase activation on resistive-breathing induced lung injury, the specific Src inhibitor PP-2 was administered prior to initiation of IRB at a dose of 1 mg/kg ip [9]. Although, PP-2 administration achieved to inhibit Src kinase activation during IRB, this inhibition was associated with a modest protective effect. In details, PP-2 administration ameliorated the increase in BAL cellularity (for both neutrophil and macrophage count) following 6 h IRB. In contrast, lung permeability, as assessed by the indirect marker of total protein level in BAL and total lung injury score were not influenced by Src inhibition. Also, the increase in tissue elasticity following 6 h of IRB was partially reversed.

In agreement with our data, Src activation has been shown to mediate neutrophil infiltration following intratracheal LPS administration [13]. PP2 pretreatment failed to reduce MIP-2a levels in lung tissue following IRB, indicating that the effect of PP-2 was independent of an IL-8 (analogue of MIP-2a) mediated mechanism. Although this finding is in contrast with previous studies, involving LPS [24] and ischemia/reperfusion [25]–induced lung injury, showing reduced chemokine expression (MIP-2a and GRO/KC, respectively) following Src inhibition, Src may also mediate neutrophil infiltration through affecting adhesion molecules, such as ICAM-1 [26] and integrin-β3 [13].

Src kinase inhibition, on the other hand, failed to attenuate the increase in total protein levels in BAL fluid, an indirect index of the integrity of the blood-gas barrier after 6 h of IRB. This is in contrast with previous studies showing that Src inhibition protects lung permeability, against high pressure-induced [12] and LPS-induced lung injury [13]. An interesting observation from our data, is that although Src inhibition prevented the influx of leukocytes in the lung, it did not protect against the increase in lung permeability. Thus, the effect of negative intrathoracic pressure during IRB on lung permeability is direct and independent of the concurrent inflammation, at least in the early phase. In accordance to this finding of our study, a rapid increase in lung permeability has been shown after high pressure ventilation, preceding the upregulation of cytokine expression [27].

An interesting finding of our study was that at 3 h of IRB, blocking Src activation attenuated also the activation of ERK1/2 through phosphorylation, suggesting an early Src-mediated ERK1/2 activation during IRB. Activation of ERK1/2 through phosphorylation has been previously reported in vivo, when the lung was subjected to increased mechanical stress [28]. Indeed, in vitro studies in human pulmonary epithelial cells and bovine vascular endothelial cells showed that Src kinase mediates mechanical stress-induced ERK phosphorylation [11, 29]. In contrast, in our model, Src inhibition failed to block ERK1/2 activation at 6 h of IRB, suggesting a late Src-independent ERK1/2 activation mechanism during IRB. Although, the underlying mechanism of the late (6 h) ERK1/2 activation cannot be adressed by the present study, in vitro ERK phosphorylation following mechanical deformation of lung resident cells is shown to be mediated by various pathways, including oxidative stress [30], EGFR [31], endothelial microparticle shedding [32] and TRPV4 calcium channel activation [33]. In the literature a variable response of “mechanotransductive” pathways is common. For example, Chaturvedi LS et al., 2007 have shown that mechanical deformation of intestinal epithelial cells on collagen leads to ERK1/2 activation by a Src-dependent pathway [34], whereas the same research group has shown that ERK1/2 activation is Src-independent, when stretch was applied on fibronectin, under the same conditions and cell type, despite Src being also activated [35].

Since Src inhibition exerted only a partial protective effect against IRB-induced acute lung injury and was associated with a transient attenuation of ERK1/2 activation (at 3 but not at 6 h of IRB), we further explored the potential role of sustained ERK1/2 inhibition in resistive breathing-induced lung injury, using the ERK1/2 activation inhibitor, PD98059. ERK inhibition attenuated both macrophage and neutrophil infiltration following IRB and reduced IL-1β levels in lung tissue. Moreover, ERK1/2 inhibition prevented the increase in lung permeability, as assessed by the evans blue dye extravasation technique and partly reversed the increase in tissue elasticity. In agreement with our data, inhibition of ERK has been found to protect against LPS-induced lung injury in both mice [36] and rats [37]. Also, in vitro, ERK1/2 activation contributes to increased permeability of rat alveolar epithelial cell monolayer following cyclic stretch [38]. As with Src kinase inhibition, blocking of ERK activation during IRB failed to reduced MIP-2a levels, despite preventing the influx of neutrophils in the alveolar space. ERK inhibition during high pressure ventilation in isolated perfused mouse lungs also did not affect the increase of MIP-2a levels in perfusate [39], although in LPS-induced lung injury MIP-2a expression was significantly reduced by ERK inhibition [36].

### Critique of methods

A limitation of our study is the use of 100% of oxygen to prevent hypoxemia during IRB. Although hyperoxia is found to induce lung injury in a time scale much larger than our time phrame [40] and our control animals have also received 100% oxygen, a possible synergy between hyperoxia and resistive breathing can not be completely excluded by our study [41].

The application of sole inspiratory resistive breathing in our animal model of resistive breathing is clinically relevant mainly for diseases of upper airway obstruction, such as post-obstructive pulmonary edema, obstructive sleep apnea and laryngotracheobronchitis in which the inspiratory resistance is greater than the expiratory resistance due to the extrathoracic obstruction. However, we have expanded our resistive breathing rat model to include not only solely inspiratory, but solely expiratory or both inspiratory and expiratory resistances, the expiratory being higher than the respective inspiratory resistance to mimic the situation observed in obstructive airway diseases like asthma and COPD [8]. The inflammatory and injurious response in the lung in the combined inspiratory and expiratory resistive breathing model (which is much closer to asthma or COPD than solely inspiratory resistive breathing) was very similar to the inflammatory and injurious process of the solely inspiratory resistive breathing, which further validates the results of the current study (in which we have used only inspiratory resistive breathing).

The activation of the Src kinase early after the initiation of IRB, a known intracellular sensor of mechanical stress [10], implies that lung parenchyma is indeed subjected to increased mechanical deformation during resistive breathing. Breathing through increased inspiratory resistance results in raised transmular alveolar-capillary presssure gradient, due to the large negative intrathoracic pressure and by raising capillary pressure through increased venous return and increased left heart afterload [42]. Our data support the theory that negative pressure pulmonary edema is a form of high permeability edema [42–44] and are in accordance with previous studies reporting increased levels of circulating markers of lung injury in obstructive sleep apnea patients [45, 46].

Although obstructive diseases of peripheral airways, such as asthma and COPD, are mainly characterized by the increase of expiratory resistance, inspiratory resistance is also elevated [47, 48]. Indeed, increased airway resistance is a major part of the increased inspiratory work of breathing in intubated COPD patients following an acute exacerbation [49]. Thus, resistive breathing and negative intrathoracic pressures in these patients are needed not only to overcome the intrinsic positive airway pressure but also the increased airway resistance and our findings may also apply to obstructive diseases of peripheral airways, such as asthma and COPD. Indeed, the inspiratory load chosen in this study resembles the resistive loading present during severe stabe airway obstructive diseases and/or exacerbations [1, 6]. For example, in children with asthma exacerbation, esophageal pressure during tidal breathing was measured at ~70% of maximum pressure [50]. In COPD patients that required invasive mechanical ventilation due to disease severity, a negative intrathoracic pressure of ~62% of maximum was measured [51].

The role of Src kinase and ERK in obstructive airway diseases is under investigation [52, 53]. For example, Geraghty et al. found that cigarette smoke exposure in mice was followed by an activation of Src kinase in the lung that mediated subsequent ERK activation and induction of pulmonary inflammation [including metalloproteinases (MMP)-9 and −12 expression] and airspace enlargement [54]. Increased ERK activity was also detected in lung samples of emphysematous patients that were not currently smoking, denoting that ERK activation was associated with the presence of pulmonary emphysema per se [55]. Moreover, ERK inhibition had an anti-inflammatory effect in a mouse model of allergic asthma [56]. In accordance, stretching of lung parenchymal strips further augmented the phosphorylation of ERK1/2, compared to basal levels, in a murine model of asthma [57].

## Conclusions

Our data suggest that both Src kinase and ERK are activated in the lung following inspiratory resistive breathing and ERK1/2 activation is partially mediated by Src kinase. Mainly ERK1/2 activation and to a lesser degree Src kinase activation contribute to IRB-induced acute lung injury. Thus, the results of our study raise the intriguing possibility that resistive breathing is an independent stimulus for Src kinase and ERK activation in obstructive airway diseases, especially during exacerbations.

## Abbreviations

BAL: Bronchoalveolar lavage
COPD: Chronic obstructive pulmonary disease
ERK: Extracellular signal-regulated kinase
FOT: Forced oscillation technique
IL-1b: Interleukin-1b
IRB: Inspiratory resistive breathing
MIP-2a: Macrophage inflammatory protein-2a
